# An Unusual Combination of Injuries Involving Lateral Dislocation of the First and Fifth Metatarsophalangeal Joints Along With Fractures of the Other Toes on the Same Foot: A Report of a Rare Case

**DOI:** 10.7759/cureus.95287

**Published:** 2025-10-24

**Authors:** Mohamed Jiddi, Georges F Bassil, Zied Missaoui

**Affiliations:** 1 Orthopedic Surgery, Grand Hôpital de l'Est Francilien, Meaux, FRA

**Keywords:** dislocation, foot, fracture, hallux, lateral, metatarsophalangeal, toe

## Abstract

Dislocations of the metatarsophalangeal (MTP) joints following trauma are uncommon and often underreported. Most documented cases involve the first MTP joint, or occasionally one or two of the lesser MTP joints, with the direction of dislocation predominantly dorsal. The rarity of these injuries, particularly those affecting multiple joints or exhibiting atypical patterns, poses challenges in both diagnosis and management. In some instances, diagnosis is difficult without imaging. Prompt reduction is essential to prevent neurovascular complications. MTP joint dislocations can be reduced using either closed or open methods, depending on the presence of soft tissue interposition. This case describes a 23-year-old patient who sustained a rare combination of pure lateral dislocations of the first and fifth MTP joints, along with multiple metatarsal fractures, following a road traffic accident. Reduction, immobilization, and rehabilitation led to a favorable outcome. The aim of this article is to describe our treatment strategy and to provide an overview of the existing literature on these unusual injuries.

## Introduction

First metatarsophalangeal (MTP) dislocations are rare, with the dorsal type being the most common. While an isolated injury may result in MTP joint dislocation, it is often accompanied by other traumas. Numerous variations of this injury have been described due to differing mechanisms and possible anatomical disruptions. Despite many attempts, the Jahss classification does not encompass all patterns of these injuries, as it is limited to dorsal MTP dislocations and their relationship with the sesamoid complex [1,2]. To address all variations of MTP joint dislocation, Zrig et al. proposed a more comprehensive classification system based on the type of displacement and the feasibility of open or closed reduction [3].

Regardless of the dislocation type or the presence of associated lesions, prompt reduction is essential due to the risk of neurovascular injury. Clinically, the affected toe may appear shortened or deformed. Although the skin is usually intact, soft tissue interposition can impede closed reduction, necessitating open techniques. Without radiographic imaging, the injury can be difficult to diagnose because of preexisting deformities.

We present a case involving a 23-year-old patient who sustained an unusual combination of multiple injuries to the same foot following a road traffic accident. This included a rare pure lateral dislocation of the first and fifth MTP joints, associated with neck fractures of the second, third, and fourth metatarsals. He was treated with closed reduction and immobilization in a posterior splint for four weeks, followed by a functional rehabilitation program, resulting in a favorable outcome.

## Case presentation

A 23-year-old male with no prior medical history was admitted to the emergency department following a road traffic accident involving a collision between two motorcycles. He sustained multiple nonsevere injuries, the most significant of which affected his right foot.

Clinical examination revealed a conscious, well-oriented patient who was unable to bear weight on the affected foot due to pain. The right foot was visibly deformed and swollen, particularly around the first and fifth toes, with a small 1 cm wound on the medial aspect of the foot and partial protrusion of the first metatarsal head. Unfortunately, no clinical photographs are available. Gentle palpation elicited intense pain over the necks of the other metatarsals, but no neurovascular deficits were observed.

**Figure 1:**
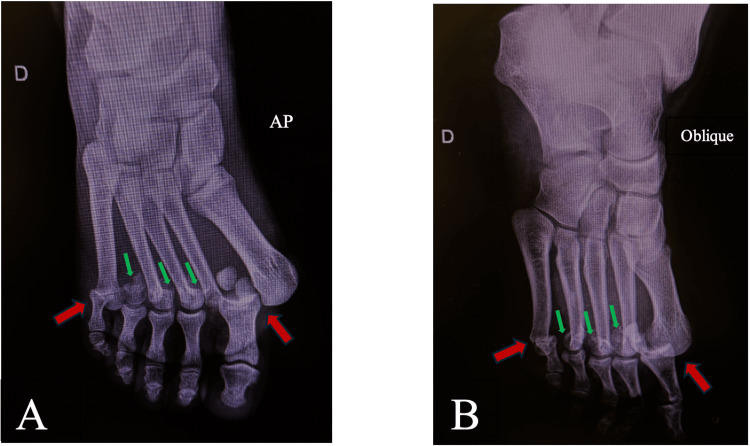
AP (A) and oblique (B) initial right foot radiographs showing lateral dislocations of the first and fifth MTP joints (red arrows) and neck fractures of the second, third, and fourth metatarsals (green arrows) MTP, metatarsophalangeal

Initial radiographs of the right foot demonstrated an unusual combination of injuries: complete lateral dislocations of the first and fifth MTP joints, associated with neck fractures of the second, third, and fourth homolateral metatarsals (Figure 1).

Given the multiple and open injuries, the patient was transferred to the operating room for wound exploration, debridement, and joint irrigation, followed by reduction of the MTP joints using simple axial traction. A soft tissue injury was identified on the medial side, including rupture of the articular capsule and medial ligament. After repair, the joints were clinically stable in both planes. Dynamic stress fluoroscopy was also performed to confirm reduction and stability.

**Figure 2:**
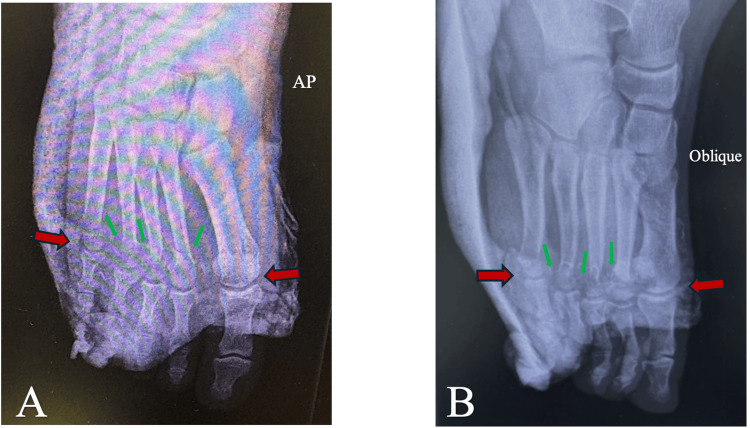
AP (A) and oblique (B) right foot radiographs after reduction of the first and fifth MTP joints (red arrows) and reduction of the neck fractures of the second, third, and fourth metatarsals (green arrows) MTP, metatarsophalangeal

Postoperative radiographs were obtained following orthopedic treatment with a posterior foot splint (Figure 2).

The patient received IV prophylactic antibiotics for 48 hours, oral analgesics, and enoxaparin to prevent venous thromboembolism. Postsurgical clinical monitoring of wound healing was performed during the initial days of hospitalization to prevent infectious complications. An early follow-up consultation was scheduled one week later to assess the progress of scar healing.

At the one-month follow-up, the splint was removed, and the patient began foot rehabilitation three to four times per week to improve strength and mobility, with gradual progression to weight-bearing activities. During the early stages of rehabilitation, the patient reported mild discomfort during active mobilization of the great toe and when walking long distances, but he did not experience any sensation of instability.

At 12 months follow-up, the patient had returned to his normal daily activities, wearing regular shoes without pain. He also regained full active and passive range of motion of his right forefoot. Overall, the patient achieved favorable clinical and radiological outcomes without any significant complications.

## Discussion

After a thorough review of the literature and major databases, we identified only one case identical to ours, published by Ibn El Kadi in 2013 [4]. This case involved lateral dislocations of the first and fifth MTP joints, accompanied by fractures of the second, third, and fourth metatarsal necks. Given the rarity of this condition and the complex pattern of injuries, it is both reasonable and appropriate to focus on the rarest lesion, lateral dislocation of the first ray MTP joint, which presents a significant therapeutic challenge. In contrast, the other associated injuries are well-documented, with standardized treatment protocols and generally favorable outcomes.

Dislocation of the first toe MTP joint was first described nearly a century ago by Mouchet in 1931 [5]. In the early 1980s, Jahss provided a more detailed understanding of this injury and introduced a classification system for dorsal MTP dislocations, the most common type. His classification, based on the condition of the sesamoid complex, remains a valuable tool for determining whether closed or open reduction is appropriate [1,2].

However, the emergence of cases with different mechanisms and patterns of displacement has highlighted the limitations of Jahss’s classification, which is restricted to three types of dorsal MTP dislocations. It is therefore incomplete and insufficient for guiding optimal treatment in certain cases, particularly lateral dislocations. To address this gap and to encompass the various types of first MTP joint dislocations based on the displacement of the first phalanx along with their respective treatment approaches, Zrig et al. [3] introduced a more inclusive classification system in 2017. This revised system categorizes first ray MTP joint dislocations into three distinct types (Table 1).

**Table 1:** Summary of Zrig et al.’s classification of MTP dislocations MTP, metatarsophalangeal Source: [3]

Type	Description	Subtype	Details	Strategy
Type I	Dorsal dislocation of the first MTP joint	IA	The intersesamoid ligament is intact; the sesamoid complex is not dislocated	Closed reduction possible
		IB	The intersesamoid ligament is intact; the sesamoid complex is dislocated over the metatarsal neck	Open reduction necessary
		IC	Real discontinuity of the intersesamoid ligament (tearing or avulsion) is present	Closed reduction possible
Type II	Lateral and medial dislocations of the first MTP joint	IIA	Pure lateral dislocation	Closed reduction possible
		IIB	Dorsolateral or dorsomedial dislocation	Closed reduction possible
Type III	Plantar dislocation of the first MTP joint	-	-	Closed reduction possible

According to the updated classification, the dislocation observed in our case corresponds to a type IIA, characterized by a pure lateral displacement. In this context, the sesamoid complex has limited clinical significance, given the minimal risk of incarceration. These observations support the conclusions of Zrig et al. in their original work, suggesting that closed reduction can be effectively employed for this specific type of dislocation. This contrasts with certain dorsal MTP joint dislocations, where soft tissue entrapment often necessitates open surgical intervention.

In the closed reduction technique, axial traction is typically applied, sometimes with a directed medial force on the proximal phalanx, while the foot is stabilized. When successful, closed reduction immediately corrects the visible deformity and restores proper joint alignment [6,7].

In our case, the reduction was notably straightforward. The patient was fully anesthetized, ensuring complete muscle relaxation. The medial wound around the MTP joint and disruption of the intrinsic stabilizing structures facilitated precise reduction and allowed thorough evaluation of joint stability after the procedure. Stability was subsequently restored by carefully repairing and suturing the capsule and medial ligament. In cases of open MTP dislocation, meticulous repair of the medial soft tissues is essential for restoring and maintaining joint stability, particularly in the presence of valgus instability, a point also emphasized by Vosoughi and Rippstein [8].

To better understand this uncommon injury, we conducted a comprehensive review of published cases, focusing on classification, associated lesions, and treatment strategies (Table 2).

**Table 2:** Summary of documented lateral first MTP joint dislocation cases and their characteristics IP, interphalangeal; MTP, metatarsophalangeal

Author(s)	Year	Age (years)	Gender	Type	Associated injury	Treatment
Henderson et al. [9]	1986	19	Male	Open	Big toe IP joint dislocation	Open reduction + conservative treatment
Gale [10]	1991	20	Male	Closed	Neck fractures of the second, third, and fourth metatarsals	Closed reduction + orthopedic treatment
Bousselmame et al. [11]	2001	24	Male	Open	Head fracture of the second metatarsal	Closed reduction + orthopedic treatment
Kasmaoui et al. [12]	2003	28	Male	Closed	Neck fractures of the second and third metatarsals and Lisfranc joint dislocation	Open reduction + internal fixation
Piétu [13]	2005	23	Female	Closed	-	Closed reduction + orthopedic treatment
	2005	25	Male	Open	Fracture dislocation of the second MTP joint; dislocation of the third MTP joint	Open reduction + internal fixation
Yun et al. [14]	2008	38	Male	Closed	Avulsed bone fragment of the proximal phalanx of the big toe	Open reduction + internal fixation
Ibn El Kadi [4]	2013	34	Male	Open	MTP dislocation of the fifth toe and neck fractures of the second, third, and fourth toes	Open reduction + internal fixation
Vosoughi and Rippstein [8]	2017	44	Male	Open	Neck fracture of the second metatarsal and base fractures of the third and fourth metatarsals	Open reduction + orthopedic treatment
Concha et al. [15]	2022	28	Male	Open	MTP dislocations of the second, third, fourth, and fifth toes	Open reduction + orthopedic treatment
Our case	2025	23	Male	Open	MTP dislocation of the fifth toe and neck fractures of the second, third, and fourth toes	Closed reduction + orthopedic treatment

Effective postoperative care and adherence to a structured rehabilitation program are essential to minimize local complications and optimize functional outcomes. The prognosis of first MTP joint dislocations is closely linked to the type of dislocation and the adequacy of initial management. While many patients achieve satisfactory outcomes, several complications have been reported. Short-term complications may include instability, joint stiffness, or infection. Long-term complications reported in the literature include post-traumatic hallux rigidus, osteoarthritis, metatarsal head osteonecrosis, complex regional pain syndrome, and sesamoid pseudarthrosis.

In our case, at one-year follow-up, no acute or mid-term complications were observed. The patient will continue to be monitored carefully to identify any potential long-term issues.

## Conclusions

This rare case of lateral dislocation of the first MTP joint, combined with multiple metatarsal fractures, highlights the complexity of forefoot trauma. The need for open reduction due to soft tissue interposition underscores the importance of early diagnosis and timely surgical intervention when closed reduction is unsuccessful.

Repair of the medial soft tissues, particularly in the presence of valgus instability, was essential for restoring joint stability. The favorable outcome in this case supports the relevance of the Zrig classification in guiding treatment decisions and reinforces the value of a tailored surgical approach in managing complex MTP dislocations.

## References

[1] Jahss MH (1980). Traumatic dislocations of the first metatarsophalangeal joint. Foot Ankle.

[2] Jahss MH (2006). Classic article: foot & ankle 1:15, 1980 traumatic dislocations of the first metatarsophalangeal joint. Foot Ankle Int.

[3] Zrig M, Othman Y, Bellaaj Z, Koubaa M, Abid A (2017). Dislocation of the first metatarsophalangeal joint: a case report and suggested classification system. J Foot Ankle Surg.

[4] Ibn El Kadi K, Saliou S, Benabid M (2013). Convergent lateral dislocation of the great and the fifth toes associated with ipsilateral fracture of the other toes: report of a rare lesion association [Article in French]. Med Chir Pied.

[5] Mouchet A (1931). Two cases of complete dorsal dislocation of the big toe with sesamoid injuries [Article in French]. Rev Orthop.

[6] De Palma L, Santucci A, Marinelli M (2001). Traumatic dislocation of metatarsophalangeal joints: report of three different cases. Foot Ankle Surg.

[7] Chisholm M (1914). Injuries of the foot: with a new method of reducing dislocation of the big toe. Can Med Assoc J.

[8] Vosoughi AR, Rippstein PF (2017). Rare lateral dislocation of the first metatarsophalangeal joint: a case report and review of the literature. J Foot Ankle Surg.

[9] Henderson CE, Denno GJ (1986). Simultaneous open dislocation of the metatarsophalangeal and interphalangeal joints of the hallux: a case report. Foot Ankle.

[10] Gale DW (1991). Lateral dislocation of the first metatarsophalangeal joint, a radiographic indicator of reducibility. Injury.

[11] Bousselmame N, Rachid K, Lazrak K, Galuia F, Taobane H, Moulay I (2001). New varieties of lateral metatarsophalangeal dislocations of the great toe [Article in French]. Rev Chir Orthop Reparatrice Appar Mot.

[12] Kasmaoui EH, Bousselmame N, Bencheba D, Boussouga M, Lazrek K, Taobane H (2003). The floating metatarsal. A rare traumatic injury. Acta Orthop Belg.

[13] Piétu G (2005). Lateral dislocation of the first metatarsophalangeal joint: report of two cases. J Trauma.

[14] Yun YS, Kim YM, Kim KC, Kim PS (2008). Lateral dislocation of the first metatarsophalangeal joint: a case report. J Korean Fract Soc.

[15] Concha JM, González H, Concha C, Rosas LA (2022). Uncommon case of traumatic dislocation of all five metatarsophalangeal joints: results of treatment after 36 months. Foot Ankle Surg.

